# Elevated cellular accumulation of endogenous and exogenous CoQ by altered intracellular trafficking

**DOI:** 10.1016/j.jbc.2025.110878

**Published:** 2025-11-04

**Authors:** Ying Wang, Siegfried Hekimi

**Affiliations:** Department of Biology, McGill University, Montreal, Quebec, Canada

**Keywords:** Coenzyme Q, CoQ, ubiquinone, UQ, CoQ turnover, Coenzyme Q deficiency, iron, lysosome, paraquat

## Abstract

Coenzyme Q (CoQ) is produced in the inner membrane of mitochondria, from where it is transported to other cellular membranes. Cellular CoQ levels drop when its synthesis is interrupted, indicating that it can be degraded or eliminated in some way by currently still uncharacterized mechanisms. Low cellular iron availability has been found to lower CoQ levels, at least in part by inhibiting the action of the CoQ biosynthetic enzyme COQ7. These findings prompted us to test the effect of elevated intracellular iron content on CoQ levels. In the mouse macrophage cell line RAW264.7, we found that supplementation with ferrous ions (Fe^2+^) boosts CoQ levels rapidly and reversibly. Iron loading also increases the cellular accumulation of exogenous CoQ_10_ provided in the media. N-acetyl cysteine significantly attenuates the elevation of CoQ levels by iron, suggesting that the effect of iron is mediated by a redox mechanism, although overall cellular reactive oxygen species (ROS) levels were not affected. Treating RAW264.7 cells with the ROS-generator paraquat also dramatically increases CoQ, further pointing to a redox mechanism. No effect on the abundance of several COQ proteins was observed after iron or paraquat treatment, indicating that their effect on CoQ levels is unlikely to arise from altered mitochondrial CoQ synthesis. In contrast, we observed that targeting lysosome function also affects CoQ levels, suggesting that the effects we observe relate to degradation and/or recycling. Our study suggests that targeting these mechanisms could allow for new therapeutic options to boost cellular CoQ levels in patients.

Coenzyme Q (CoQ), also known as ubiquinone, is an ancient and highly conserved lipophilic molecule, composed of a redox-active head group and an extremely hydrophobic isoprenoid tail whose length is species-specific. The benzoquinone head, synthesized from tyrosine, enables the molecule to serve as an electron carrier, alternating between the reduced and the oxidized states through a semiquinone intermediate, while the polyisoprene tail, synthesized from mevalonate, serves as a lipid membrane anchor (1, 2). In CoQ nomenclature, the tail length is denoted by a numerical subscript referring to the number of isoprene subunits. Humans primarily utilize CoQ_10_, while CoQ_9_ predominates in rodents, and *Saccharomyces cerevisiae* has CoQ_6_ (3, 4). The most essential function of CoQ is to act as an electron carrier in the mitochondrial respiratory chain, transferring electrons from complex I, complex II, and several other dehydrogenases, to complex III (5, 6, 7). Moreover, CoQ—which is present in all cellular membranes—performs a variety of additional functions depending on location (8). In the plasma membrane, it is a part of the transmembrane electron transport system (PMET). In addition, the ferroptosis suppressor protein 1 (FSP1) regulates ferroptosis by acting as a CoQ oxidoreductase (9, 10, 11, 12). In the lysosome, CoQ participates in an electron transport chain, which promotes the translocation of protons across the lysosomal membrane into the lumen, thereby being essential for maintaining an acidic luminal pH (13, 14). CoQ is also widely hailed as an antioxidant that protects membrane lipids and circulatory lipoproteins against peroxidation (8, 15).

CoQ is synthesized in the inner membrane of mitochondria. Although the biosynthetic pathway of CoQ in animals has not been fully elucidated, most genes essential for this pathway, termed *COQ* genes, have been identified. The biosynthesis of CoQ relies on the formation of a multi-subunit complex of COQ proteins, termed the Q complex (16, 17, 18, 19, 20, 21). Loss of CoQ production due to mutations in *COQ* genes causes genetic CoQ deficiency, a disease that has been increasingly reported in the literature since its first description in 1989 (1, 4, 17, 22, 23, 24, 25, 26, 27, 28, 29, 30, 31). Furthermore, CoQ deficiency has been reported to be associated with various conditions that are not due to an inherited mutation in a CoQ biosynthetic gene, *e.g.*, several different types of mitochondrial disorders, cerebellar ataxia, Parkinson's disease, and aging (32, 33, 34, 35, 36, 37, 38, 39, 40).

CoQ deficiency mostly presents as a typical mitochondrial disorder but can vary greatly in both presentation and severity (17, 24, 26, 27, 28, 29, 31, 41, 42, 43, 44, 45, 46, 47). So far, oral CoQ_10_ supplementation is the only available treatment for CoQ deficiency. However, this treatment is at best inadequate due to its poor bioavailability (1, 17, 47, 48, 49, 50). To overcome the unsatisfactory outcomes of oral CoQ_10_ therapy, novel CoQ_10_ formulations have been proposed, aiming to enhance the uptake of exogenous CoQ (51, 52, 53, 54, 55, 56, 57, 58, 59, 60). However, another strategy for boosting CoQ levels could be to target the fate of endogenous CoQ. Indeed, CoQ is lost from cells when its synthesis is interrupted, indicating that it can be degraded or eliminated in some ways, but the corresponding mechanisms are largely unknown (61, 62, 63, 64). Here, we explored whether the steady state of synthesis and elimination of CoQ can be altered, as this would provide new paths for developing drugs that increase intracellular CoQ levels. We succeeded in demonstrating that cellular CoQ levels are elevated without significantly affecting synthesis following exposure to excess iron, paraquat, or compounds that inhibit lysosome function. Our findings suggest the possibility of boosting CoQ levels by modulating CoQ trafficking. Understanding the underlying mechanisms could uncover novel drug development strategies for CoQ deficiency.

## Experimental procedures

### Chemicals and antibodies

Lalistat one was purchased from Cedarlane Labs. Bafilomycin A1 (Baf-A1) was purchased from MedChemExpress. 2′,7′-dichlorodihydrofluorescein diacetate (DCFH-DA) was obtained from ThermoFisher Scientific. All other chemicals, including ferrous sulfate (FeSO_4_), paraquat (PQ) and N-Acetyl-L-cysteine (NAC), were obtained from Sigma-Aldrich. Most of the primary antibodies used in the study, including COQ3 (28051-1-AP), COQ4 (16654-1-AP), COQ7 (15083-1-AP), COQ8A (15528-1-AP), COQ9 (14874-1-AP), GAPDH (10494-1-AP), Cytochrome c (Cyt C) (0993-1-AP) and voltage-dependent anion-selective channel 1 (VDAC1)/Porin (55259-1-AP), were obtained from Proteintech. An antibody against superoxide dismutase 2 (SOD2) was obtained from Abcam (ab13533). An anti-ferritin heavy chain 1 (FTH1) (sc-376594)) antibody was obtained from Santa Cruz Biotechnology and an anti-β-actin antibody (#3700) was obtained from Cell Signalling Technology. Horseradish peroxidase (HRP)-conjugated horse anti-mouse IgG (#7076) and HRP-conjugated goat anti-rabbit IgG (#7074) were purchased from Cell Signalling Technology.

### Cell culture

All cell lines except mouse embryonic fibroblasts (MEFs) were purchased from ATCC. MEFs were generated from wild-type C57BL/6 mouse embryos as previously described (65). All cells were cultured and maintained at 37 °C in a humidified incubator with 5% CO_2_ in high-glucose Dulbecco’s Modified Eagle Medium (DMEM) supplemented with 10% fetal bovine serum (FBS) in the presence of 1% antibiotic–antimycotic solution (Wisent Bioproducts). Cell viability was measured using the resazurin assay in 96-well plates as previously described (6).

### Reactive oxygen species (ROS) measurement

Cells were seeded in clear, black-bottom 96-well plates (Corning, CLS3603) and subjected to the desired treatments. At the end of treatments, the media was removed, and the cells were washed once with phosphate-buffered saline (PBS). Then, 100 μl of fresh DMEM containing 10 μM DCFH-DA was added to the wells. After incubation for 30 min at 37 °C and 5% CO_2_, each well was washed with PBS before fluorescence was measured on a Tecan Infinite 200 microplate reader with excitation/emission wavelengths of 485/530 nm. Following fluorescence measurements, 20 μl of radioimmunoprecipitation assay (RIPA) buffer (50 mM Tris-HCl, pH 7.4, 1% NP-40, 0.5% sodium deoxycholate, 2 mM EDTA, 0.1% SDS, 150 mM NaCl) was added to each well, and protein amount was quantified by the bicinchoninic acid assay (BCA) kit (ThermoFisher Scientific).

### Determination of citrate synthase activity

Citrate synthase (CS) activity was measured as previously described (65). Briefly, cells were lysed in CelLytic MT cell lysis reagent (C3228, Sigma). The lysate containing 15 μg of total protein was added to 1 ml of reaction mixture containing 100 mM Tris (pH 8.0), 0.1 mM 5,5′-dithio-bis(2-nitrobenzoic acid); 3-carboxy-4-nitrophenyl disulphide (DTNB), 0.1% Triton X-100 and 0.1 mM acetyl-CoA. 10 μl of 0.25 mM oxaloacetate was then added to start the reaction, and absorbance at 412 nm was measured every 15 s for 3 min. The rate was calculated from the linear range and normalized to total protein. The enzymatic activity was calculated using the molar extinction coefficient of 5,5′-dithiobis-(2-nitrobenzoic acid) (13.6 mM^−1^ cm^−1^).

### Mitochondrial isolation

Mitochondria were isolated using a differential centrifugation method (51). In brief, cells were homogenized with a motor-driven Teflon Potter-Elvehjem homogenizer (Wheatone) in a buffer consisting of 250 mM sucrose, 1 mM ethylenediaminetetraacetic acid (EDTA) and 10 mM 4- (2-hydroxyethyl) -1-piperazineethanesulfonic acid (HEPES) buffer, pH 7.4. The resulting homogenate was centrifuged at 700*g* for 10 min, and the resulting supernatant was collected and centrifuged at 10,000*g* for 10 min (Beckman Coulter Avanti J-25). The mitochondrial pellet was washed once before being resuspended in the same isolation buffer and stored at −80 °C before analysis.

### CoQ extraction and quantification

CoQ quantification was carried out by high-performance liquid chromatography (HPLC) as previously described (42, 64, 66). Briefly, cells were lysed in RIPA buffer, and CoQ was extracted using a hexane: ethanol (5:2, v/v) mixture. Chromatographic separation was carried out on an Agilent 1260 Infinity LC system (Agilent Technologies), using a reverse-phase C18 column (2.1 × 50 mm, 1.8 μm, Agilent) at a flow rate of 0.3 ml/min. The mobile phase contained 70% methanol and 30% ethanol (vol/vol). The UV wavelength of 275 nm was used to detect CoQ in the eluent. Peak identities and CoQ amounts were established by comparison with the peaks obtained from CoQ standards. The final quantification of CoQ was normalized to the amount of protein measured with a BCA assay.

### Western blotting

Cells were lysed in RIPA buffer supplemented with protease/phosphatase inhibitor cocktail (#5872, Cell Signalling Technology). 40 μg (for COQ or LC3 detection) or 90 μg (for FTH1) of protein were separated on a 12% SDS-PAGE gel and transferred to a nitrocellulose membrane (Bio-Rad). The membranes were probed with the primary antibodies at 4 °C overnight, followed by incubation with appropriate HRP-conjugated secondary antibodies for 1 h (Cell Signalling Technology). Blots were developed with ECL reagent (CCH345, Froggabio) and exposed to X-ray film to visualize bands of interest. Blot quantitative analysis was done with myImageAnalysis Software (version 2.0; Thermo Fisher Scientific).

### Protein identification and quantification

RAW264.7 cells were treated with FeSO_4_ (0.25 mM) or paraquat (PQ) (30 μM) with or without N-acetylcysteine (NAC) (7.5 mM) for 48 h, with three replicates in each group. The protein concentration was determined using a BCA protein detection kit (ThermoFisher Scientific) after lysis using RIPA buffer supplemented with protease and phosphatase inhibitor cocktails ((#5872, Cell Signalling Technology). 60 μg of total protein from each sample was sent to the Proteomics and Molecular Analysis Platform at the Research Institute of the McGill University Health Centre (RI-MUHC) for LC-MS proteomics analysis. Briefly, for each sample, protein lysates were resolved in a stacking gel band, reduced with dithiothreitol, alkylated with iodoacetic acid, and digested with trypsin. Extracted peptides were re-solubilized in 0.1% aqueous formic acid and loaded onto an Acclaim PepMap precolumn (75 μM ID × 2 cm C18 3 μM beads) and then onto an Acclaim PepMap EASY-Spray analytical column (75 μM × 15 cm with 2 μM C18 beads). The peptides were separated using a Dionex UltiMate 3000 UHPLC at 250 nl/min with a gradient of 2%–35% organic (0.1% formic acid in acetonitrile) over 3 h. Peptides were analyzed using a Thermo Orbitrap Fusion mass spectrometer operating at 120,000 resolutions (FWHM in MS1) with HCD sequencing (15,000 resolution) at top speed for all peptides with a charge of 2^+^ or greater. The raw data were converted into a ∗.mgf format (Mascot generic format) for searching using the Mascot 2.6.2 search engine (Matrix Science) against mouse protein sequences from UniProt. Scaffold (version 5.3,3, Proteome Software Inc.) was used to validate MS/MS-based peptide and protein identifications. Peptide identifications were accepted over 95.0% probability, and protein identifications were accepted if they could be established at greater than 99.0% probability and contained at least one identified unique peptide. Quantitative method was set to total spectra, and statistical tests were performed using an ANOVA with a Benjamini-Hochberg multiple test correction at a significance level set to 0.05.

### Statistical analysis

Statistical analysis was carried out with GraphPad Prism version 10.4.2. Data are shown as means ± SEM unless otherwise specified. Means were compared between groups using an unpaired two-tailed *t* test or one- or two-way ANOVA, as appropriate and indicated in the figure legends. *p* < 0.05 was accepted to indicate statistical significance.

## Results

### Iron loading increases cellular CoQ levels

**Figure 1:**
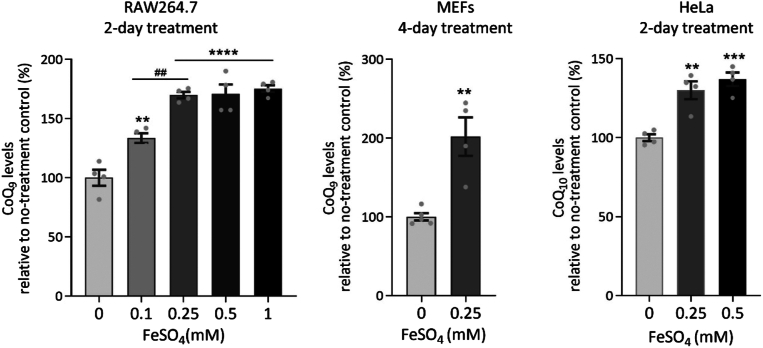
**Iron supplementation increases cellular levels of CoQ.** Data are expressed as percentages relative to no-treatment controls set to 100%. Bars represent mean ± SEM (n = 3–4 per group). ∗*p* < 0.05, ∗∗*p* < 0.01, ∗∗∗*p* < 0.001, and ∗∗∗∗*p* < 0.0001 vs. no-treatment control; ^##^*p* < 0.01 between different dosage groups (one-way ANOVA with Sidak's *post hoc* test or *t* test). Refer to Table S1 for the measured CoQ concentrations in ng/mg protein for all figures presenting CoQ levels as percentages of no-treatment controls.

In a previous study, we demonstrated that lowering cellular iron availability lowers CoQ levels and elevates the levels of the biosynthetic intermediate demethoxyubiquinone (DMQ) (61, 62). This happens because COQ7, the enzyme responsible for the penultimate step of CoQ biosynthesis, which converts DMQ into 5-hydroxyUQ, is a di-iron carboxylate hydroxylase whose prosthetic iron is sensitive to iron withdrawal (67). Iron loss from the enzyme or replacement by other metals, therefore, results in reduced CoQ production and lower cellular CoQ levels (61, 62, 63). These findings prompted us to test the effect of high iron levels on CoQ levels. For this, we delivered iron in the form of FeSO_4_, which results in increased availability of ferrous iron (Fe^2+^) in the cell. Working with a mouse macrophage cell line (RAW264.7), mouse embryonic fibroblasts (MEFs), and human HeLa cells, we found that iron supplementation boosts cellular CoQ levels in all three cell types (Fig. 1). Among those cell types, we found that the RAW264.7 mouse macrophages are particularly sensitive to Fe^2+^ treatment, as previously suggested by the finding that iron rapidly reverses manganese-induced inhibition of CoQ synthesis in these cells (62). This is why most subsequent experiments were carried out with RAW264.7 cells.

**Figure 2:**
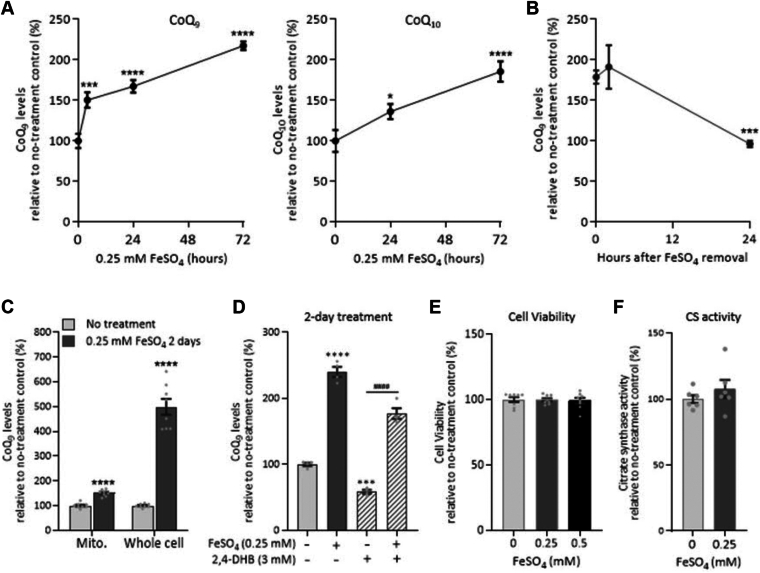
**Effect of iron supplementation on cellular levels of CoQ is rapid and reversible.***A*, time course of changes in CoQ levels after treatment with FeSO_4_. *B*, changes in CoQ levels after FeSO_4_ withdrawal from the culture medium following 24h exposure. *C*, comparison of the effects of FeSO_4_ on mitochondrial and total cellular CoQ levels. *D*, effect of iron treatment on CoQ levels in cells co-treated with 2,4-dihydroxybenzoic acid (2,4-DHB). 2,4-DHB inhibits CoQ biosynthesis. Under such a condition, iron treatment still leads to an elevation of total cellular CoQ. *E*, no change in cell viability was observed after 2 days of treatment with FeSO_4_. *F*, no change in citrate synthase activity was observed after 2 days of treatment with FeSO_4_. Data are expressed as percentages relative to no-treatment controls set to 100%. Values are mean ± SEM (n = 3–4 per group). ∗*p* < 0.05, ∗∗∗*p* < 0.001, and ∗∗∗∗*p* < 0.0001 vs. no-treatment control, and ^*####*^*p* < 0.0001 between indicated treatment groups (one-way ANOVA with Sidak's *post hoc* test or *t* test).

We observed that the effect of excess iron on CoQ level was dose- and time-dependent (Figs. 1 and 2*A*). In RAW264.7 cells, the main CoQ species is CoQ_9_, with CoQ_10_ being the minor species. We observed an increase in both CoQ_9_ and CoQ_10_ levels after Fe^2+^ exposure (Figs. 2*A* and S1). The effect was already visible after 4 h of iron supplementation (Fig. 2*A*) and was reversible, as shown in Figure 2*B*, where the elevated CoQ_9_ level observed after 24 h of FeSO_4_ treatment returned to normal within 24 h of FeSO_4_ removal from the culture medium.

As all committed steps of CoQ biosynthesis are carried out in the mitochondria (4, 7, 16, 17), we also determined whether CoQ levels were affected by iron (at 0.25 mM) in a mitochondria-enriched fraction (Fig. 2*C*). Interestingly, the magnitude of the change in mitochondrial CoQ levels was much smaller than what was observed in whole cell lysates, a first indication that the effect of iron supplementation on CoQ level is likely not due to increased CoQ biosynthesis but to a new equilibrium integrating mitochondrial synthesis, intracellular distribution, recycling, and elimination. We have also examined the mitochondrial respiration of the iron-loaded RAW 264.7 cells. Among the three doses tested (0.1 mM, 0.25 mM and 0.5 mM), which were found to increase total cellular CoQ levels after 2 days of treatment (Fig. 1*A*), 0.25 mM FeSO_4_ showed no significant effect on basal respiration but decreased the FCCP-uncoupled (maximal) respiration rate, while 0.5 mM decreased both basal and uncoupled respiration rates (Fig. S2). Mitochondria require iron primarily for synthesizing heme and iron-sulfur clusters; however, surplus iron can lead to mitochondrial iron overload, resulting in elevated levels of ROS and ultimately impaired mitochondrial function (68). Additionally, iron overload in macrophages may reduce the expression of electron transport chain components through down-regulating glia maturation factor-γ (GMFG) (69). Interestingly, 0.1 mM FeSO_4_ showed a slight enhancing effect on basal respiration, which may be related to the elevation of CoQ levels. We further examined the effect of iron on CoQ levels under a CoQ-deficit state induced by exposure to 2,4-dihydroxybenzoic acid (2,4-DHB). 2,4-DHB is a structural analogue of 4-hydroxybenzoic acid (4-HB), the immediate precursor of the CoQ head group. 2,4-DHB inhibits CoQ biosynthesis by competing with the natural CoQ head group precursor (64, 70, 71). Under this biosynthesis-impaired condition, iron exposure still led to an elevation of cellular total CoQ (Fig. 2*D*). Lastly, it is noteworthy that iron overload can be toxic and trigger ferroptosis (72). However, at the dosages used (between 0.1 and 0.5 mM), iron supplementation had no significant effect on cell viability or mitochondrial content (Figs. 2, *D* and *E* and S3). Excess iron could promote ferroptosis (73). However, iron exposure at the tested dosages was not found to increase the cells’ susceptibility to ferroptosis induced by the glutathione peroxidase 4 (GPX4) inhibitor RSL3 (Fig. S4), indicating that iron treatment did not exacerbate oxidative stress and lipid peroxidation, the key events driving ferroptotic cell death.

### Iron loading increases the accumulation of exogenous CoQ_10_

**Figure 3:**
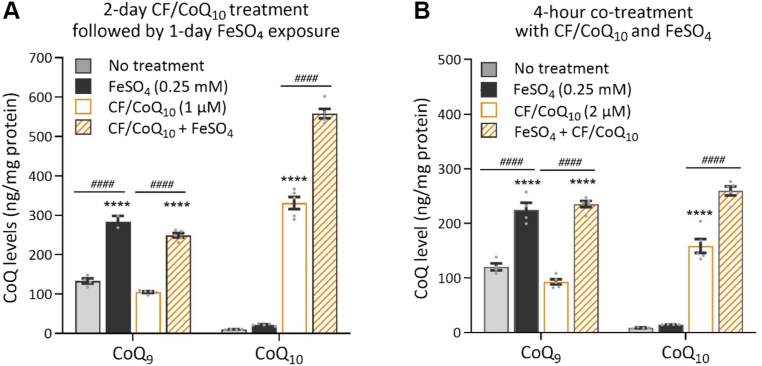
**Effect of iron supplementation on the accumulation of exogenous CoQ.***A*, change in CoQ levels in RAW264.7 cells after pre-treatment with CF/CoQ_10_ (1 μM of CoQ_10_ in the medium) for 2 days, followed by 1-day exposure to FeSO_4_ (0.25 mM). *B*, CoQ measurements in RAW264.7 cells after 4 h of co-treatment with CF/CoQ_10_ (2 μM of CoQ_10_ in the medium) and FeSO_4_ (0.25 mM), in comparison to single agent treatment or control. Data are shown as mean ± SEM (n = 4 per group). ∗∗∗∗*p* < 0.0001 vs no-treatment control; ^*####*^*p* < 0.0001 between the indicated groups (two-way ANOVA followed by Tukey's test).

We provided cells with exogenous CoQ_10_ and monitored its accumulation to further test the hypothesis that iron supplementation acts principally through altered CoQ trafficking. Exogenous CoQ_10_ is known to be able to enter cultured cells, accumulate there, and rescue the mitochondrial dysfunction due to endogenous CoQ deficiency, indicating that it can gain access to the mitochondrial compartment (6, 42, 74, 75, 76). To facilitate CoQ cellular uptake, we used our previously published method, in which CoQ_10_ is provided in mixed micelles with the echinocandin caspofungin (CF/CoQ_10_) (51). In Figure 3*A*, we treated RAW264.7 cells with CF/CoQ_10_ for 2 days, followed by its removal and addition of FeSO_4_ to the culture medium for 1 day. We observed that, as expected, exogenous CoQ_10_ had no effect on the level of endogenous CoQ_9_ but led to a large accumulation of CoQ_10_ in these cells. In Figures 1 and 2*A* above, we showed how treatment with iron increased the levels of both endogenous CoQ_9_ and endogenous CoQ_10_. But in the RAW264.7 cells pre-loaded with CF/CoQ_10_ and subsequently exposed to iron, CoQ_10_ levels increased beyond what was observed with either CF/CoQ_10_ or iron treatment alone, indicating a dramatically increased accumulation and retention of exogenous CoQ (Fig. 3*A*). Similar observations were made after as little as 4 h when CF/CoQ_10_ and iron were provided at the same time (co-treatment) (Fig. 3*B*). We found that only a negligible amount of CoQ_10_ remained in the medium after washout of CF/CoQ_10_ following a 1-day treatment (Fig. S5). These observations further strengthen the notion that the effect of excess iron on CoQ levels is primarily due to a change in intracellular CoQ trafficking and not an effect on biosynthesis.

### Evidence for a redox mechanism underlying the effect of iron supplementation

**Figure 4:**
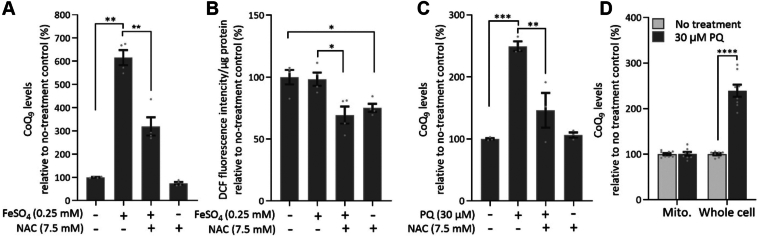
**NAC suppresses CoQ level elevation after iron or PQ exposure.***A*, CoQ measurement in RAW264.7 cells after 2-days treatment with FeSO_4_, NAC, or both. *B*, intracellular ROS detection by DCFH-DA. C. CoQ measurements in RAW264.7 cells after 2-days treatment with PQ, NAC, or both. *D*, change in CoQ levels in the whole cell or mitochondria of RAW264.7 cells after 2-days treatment with PQ. Data were expressed as percentages relative to no-treatment controls set to 100%. Bars represent means ± SEM (n = 3–4 per group). ∗*p* < 0.05, ∗∗*p* < 0.01, ∗∗∗*p* < 0.001, and ∗∗∗∗*p* < 0.0001 (one-way ANOVA in A-C and two-way ANOVA in D followed by Sidak's *post hoc* comparison test).

Iron has redox properties and can therefore affect the redox balance in the cell, including in a way that leads to the overproduction of reactive oxygen species (ROS), primarily *via* the Fenton reaction (77). To test whether the effect of iron on CoQ levels is mediated by a redox mechanism, we used N-acetyl cysteine (NAC), which boosts antioxidant defences, particularly against hydrogen peroxide (78). We found that NAC significantly suppressed the effect of iron on the accumulation of CoQ_9_ (Fig. 4*A*). We followed this up by estimating the intensity of ROS generation under these treatments, using the ROS-sensitive probe 2′,7′-dichlorodihydrofluorescein diacetate (DCFH-DA) (79). Treatment with FeSO_4_ (at 0.25 mM) did not increase ROS levels, and treatment with NAC (at 7.5 mM) decreased ROS levels equally in untreated and iron-treated cells (Fig. 4*B*). Taken together, these findings suggest that iron loading affects cellular CoQ levels by a redox mechanism, but not as a consequence of a global change in the intracellular redox state.

To further investigate whether a change in cellular redox balance affects the level of CoQ, we treated the RAW264.7 cells with the ROS-generator paraquat (PQ) at 0.03 mM for 2 days. PQ is well-known as a mitochondrial superoxide generator (80). We found that the response to PQ was similar to that to iron. PQ increased CoQ_9_ levels, with its effect being largely but not completely suppressed by NAC (Fig. 4*C*). NAC has been well demonstrated to protect against PQ-induced toxicity, primarily by replenishing glutathione levels depleted by PQ exposure (81, 82, 83). In contrast to iron treatment, which resulted in a minor increase in mitochondrial CoQ (Fig. 2*C*), no increase at all was observed with PQ treatment (Fig. 4*D*). The study with PQ reinforced the notion that a redox mechanism is involved in regulating the steady-state level of cellular CoQ, but most likely not by raising mitochondrial CoQ synthesis.

### Further lack of evidence for a mechanism involving increased mitochondrial CoQ synthesis

**Figure 5:**
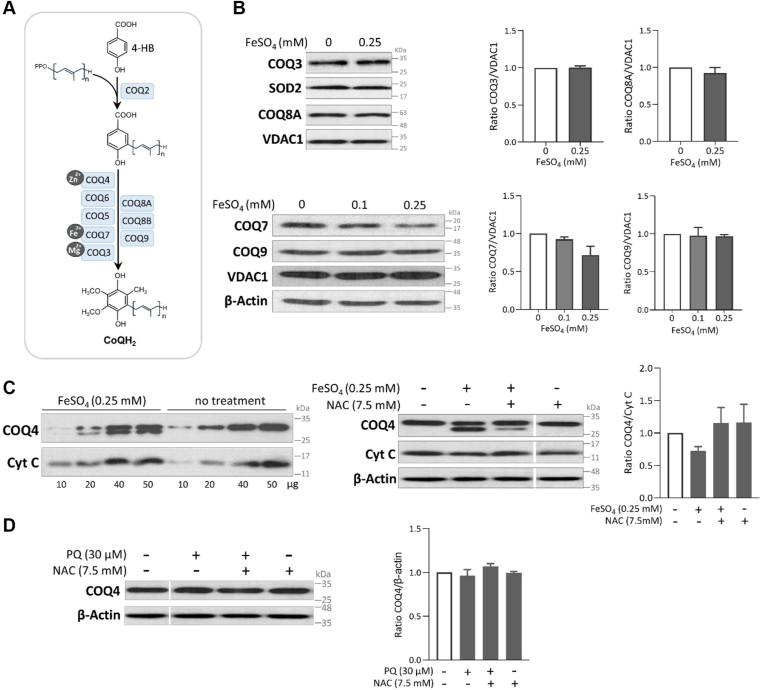
**Western blotting analysis of the levels of COQ proteins following exposure to iron or PQ.***A*, schematic of the principal components in the CoQ biosynthetic pathway. COQ2 attaches a poly-isoprenoid side chain to the CoQ ring precursor 4-HB. Subsequently, several COQ proteins catalyze sequential modifications of the aromatic ring, leading to formation of the final product. Three of the COQ proteins, COQ3, COQ4, and COQ7, require metal ions (21, 61, 63, 67). CoQH_2_ refers to ubiquinol, the reduced form of CoQ. *B*, COQ3, COQ7, COQ8A, and COQ9 levels following treatment with FeSO_4_. Except for COQ7, whose expression appears to decrease after iron treatment (*p* = 0.07), the expression of the other COQ proteins was not affected by iron treatment. *C*, Western blot analysis of COQ4. A second band of a lower molecular weight was observed in FeSO_4_-treated cells. The appearance of this band was suppressed by NAC treatment. *D*, Western blot of COQ4 in PQ-treated cells. RAW264.7 cells were collected for the analyses after 2 days of treatment. The mitochondrial proteins SOD2, VDAC1, and Cytochrome C (Cyt C), as well as cytosolic β-actin, were used as loading controls. Representative Western blot images are shown. Quantification of the bands of interest is presented in graphs as a density ratio to a loading control. Values are mean ± SEM from at least two independent experiments. In C, the graph depicts the quantification of the upper canonical COQ4 band. No significant change in the expression levels of any examined proteins was found after the indicated treatments (*t* test or one-way ANOVA followed by Dunnett's test). However, trends toward decreased levels of COQ7 and COQ4 were observed following iron treatment.

The effect of iron on exogenous CoQ loading pointed to an impact on CoQ trafficking (Fig. 3). Nonetheless, we sought to further investigate the possibility of an effect of iron and PQ treatment on mitochondrial CoQ synthesis. For this, we carried out Western blot analyses of several COQ proteins that are necessary for CoQ biosynthesis, several of which function in a multiprotein complex and require metal ion cofactors (Fig. 5*A*) (17, 18, 19, 20, 24). Neither COQ3, COQ8, nor COQ9 proteins showed any alteration of their abundance in response to iron loading (Figs. 5*B* and S6). Furthermore, the DMQ hydroxylase COQ7, which can be inhibited by iron depletion through chelation (62, 63), showed a paradoxical loss of abundance in response to increasing levels of iron (Fig. 5*B*). Although this confirms the tight relationship between iron abundance and COQ7 function, it is highly unlikely that a loss of abundance translates into higher CoQ synthesis, especially given that COQ7 carries out an enzymatic function.

In addition, an unexpected phenomenon was observed with COQ4, a CoQ biosynthesis protein which has recently been demonstrated to be required for the C1 oxidative decarboxylation of CoQ in eukaryotic cells (21, 84). Iron loading resulted in the appearance of a second COQ4-immunoreactive band with a slightly lower apparent molecular weight (Fig. 5*C*). The appearance of the band is concomitant to a decrease in the intensity of the canonical band (Fig. 5*C*). At this stage, however, we do not know if the second COQ4-immunoreactive species represents degradation, a functionally relevant splice variant, proteolytic cleavage, absence of a post-translational modification, or another form of modification. The mouse *Coq4* gene (ENSMUSG00000026798) consists of seven exons. Four protein-coding transcripts for mouse *Coq4* are listed in Ensembl and NCBI. However, none of the corresponding potential proteins have a molecular weight that would correspond to the lower COQ4 band observed following iron treatment. Interestingly, this phenomenon was largely suppressed by NAC (Fig. 5*C*). Yet, PQ treatment did not result in the appearance of an additional COQ4 band (Fig. 5*D*). Given this discrepancy, we cannot currently exclude that this phenomenon is unrelated to the effects on CoQ levels we observed with iron treatment. Additionally, it raises the possibility that the CoQ-elevating mechanisms triggered by iron and by PQ are distinct.

### Evidence for a mechanism of CoQ elimination involving lysosomal function

**Figure 6:**
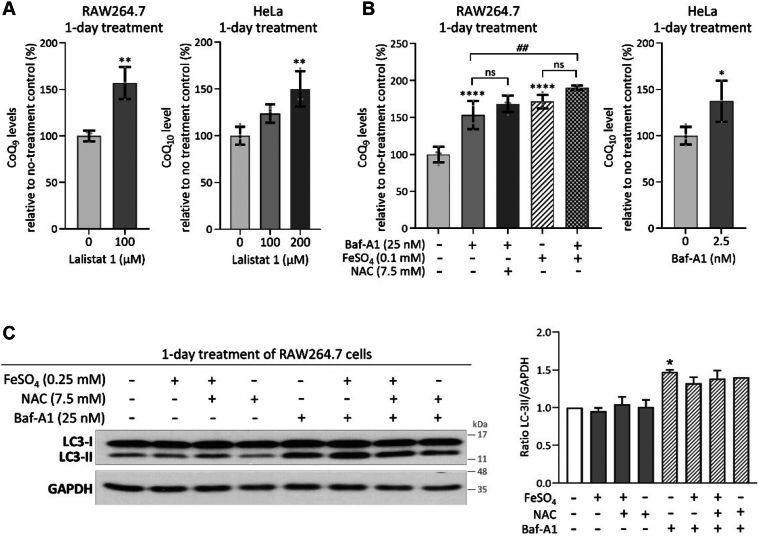
**Effects of Lalistat1and bafilomycin A1 (Baf-A1) on cellular levels of CoQ.***A*, CoQ levels were increased after Lalistat 1 treatment. *B*, changes in CoQ levels after treatment with Baf-A1. Data were expressed as percentages relative to no-treatment controls set to 100%. Bars represent means ± SEM (n = 3–4 per group). ∗*p* < 0.05, ∗∗*p* < 0.01, and ∗∗∗∗*p* < 0.0001 vs. non-treatment controls; ^##^*p* < 0.01 when the indicated groups are compared (one-way ANOVA followed by Sidak's *post hoc* comparison test). *C*, Western blot determination of LC3 protein levels. A representative image is shown. Band density ratios of LC3-II normalized to the loading control GAPDH are reported as mean ± SEM. *∗p* < 0.05 compared to the no-treatment control. Iron or NAC treatment did not increase the LC3-II levels, nor did it affect the increased conversion of LC3-I to LC3-II due to Baf-A1 co-treatment (one-way ANOVA followed by Sidak's *post hoc* test).

The mechanisms by which CoQ is lost from cells are entirely uncharacterized. It could involve elimination *via* secretion, degradation, or recycling for reuse. However, such mechanisms must exist, as endogenous CoQ is lost from cells when CoQ synthesis is stopped, even in cell types such as cardiomyocytes that do not divide or are otherwise replaced (64, 85, 86). We wondered if increases in cellular CoQ levels, such as those observed with iron and PQ, can be obtained by interfering with the degradation machinery of the cell. For this, we targeted the lysosome with Lalistat 1, a potent inhibitor of lysosomal acid lipase (LAL), which is the key enzymatic activity responsible for the degradation of neutral lipids at acidic pH (87). Of note, in mouse cells and tissues, Lalistat 1 has also been shown to inhibit cytosolic lipid hydrolases that hydrolyze neutral lipids at neutral pH (88). Strikingly, the impairment of overall lipolysis upon treatment with Lalistat 1 also increased CoQ levels in both RAW264.7 and HeLa cells (Fig. 6*A*). At this stage, we do not know whether CoQ is a target of LAL or whether the effect is more indirect by damaging some aspects of lysosomal function.

We also tested the effects of bafilomycin A1 (Baf-A1), an inhibitor of the vacuolar-type H^+^-ATPase. Baf-A1 reduces luminal acidification, thereby preventing lysosomal enzyme activation and disrupting the normal function of the lysosome (89, 90). It also blocks the fusion between autophagosomes and lysosomes, affecting the autophagic flux and could cause intracellular acidosis (91, 92). Hence, Baf-A1 treatment is expected to lead to the accumulation of autophagosomes that can’t fuse with the lysosome. This is what we observed, as revealed by the relative increase of LC3-II, the phosphatidylethanolamine-conjugated and autophagosome membrane-bound form of the microtubule-associated protein light chain 3 (LC3), compared to its cytosolic and unconjugated form (LC3-I) (Fig. 6*C*) (93, 94). In three different types of cells, RAW264.7 macrophages, HeLa cells and MEFs, we observed an elevation of CoQ levels after treatment with Baf-A1, although their sensitivity to the agent varied greatly among these cell types (Figs. 6*B* and S7*A*). NAC had no significant effect on this elevation (Fig. 6*B*), consistent with previous findings showing that NAC does not affect Baf-A1-induced lysosomal de-acidification (95). The effects of iron and Baf-1A treatment were partially additive for CoQ levels (Fig. 6*B*). Iron and NAC had no effect on autophagic flux, nor did they interfere with Baf-A1's effect on autophagy, as indicated by immunoblot analysis of LC3-I to LC3-II conversion (Fig. 6*C*). Lastly, interestingly, Baf-A1 treatment was found to induce accumulation of the CoQ biosynthetic intermediate DMQ (Fig. S7*B*), an effect not observed following iron loading (Fig. S1*A*). Together, these observations suggest that iron likely works through a mechanism that is distinct from impairment of lysosomal function.

### Proteomics analysis

**Figure 7:**
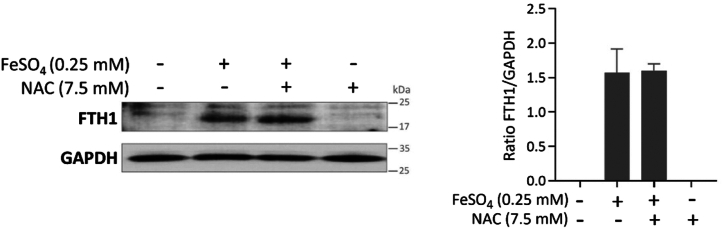
**Western Blot analysis of Ferritin Heavy Chain 1 (FTH1).** RAW264.7 cells were collected for the analyses 2 days after the indicated treatments. GAPDH was used as a loading control. The bar graph shows means ± SEM of relative FTH1 levels in the indicated samples. Data are from four independent experiments. No significant difference was found between FeSO_4_-treated cells and those simultaneously treated with NAC.

We carried out proteomics analysis of RAW264.7 cells treated with iron or PQ, with or without NAC co-treatment, compared to untreated control. A total of 3777 proteins were identified across all six sample groups (no treatment control, FeSO_4_, FeSO_4_ + NAC, PQ, PQ + NAC, and NAC), using peptide probability thresholds of 95% and protein identification threshold of 99% with a minimum of one identified unique peptide. The peptide False Discovery Rates (FDR) is 0.39%. Significant changes in protein expression detected in response to iron or PQ treatment are shown in Tables S2–S4. A total of 24 proteins were identified as differentially expressed after FeSO_4_ treatment, including ferritin light chain 1 and ferritin heavy chain, both key subunits of ferritin, which is primarily responsible for iron storage and is expected to increase following an elevation in cellular iron concentration. Notably, previous work has suggested that NAC may exhibit metal-chelating properties, including the ability to chelate iron (96, 97). However, overexpression of the ferritin subunits was also observed in the cells co-treated with FeSO_4_ and NAC (Table S2). This was confirmed by Western blot analysis, which showed a comparable elevation of ferritin heavy chain levels in FeSO_4_-treated cells and in cells treated simultaneously with NAC (Fig. 7), but not in cells exposed to PQ (Fig. S8). These suggest that the way NAC inhibits CoQ elevation caused by iron exposure is unlikely to involve direct regulation of cellular iron levels, although we cannot completely rule out the possibility of partial quenching of iron by NAC. Aconitase is another protein that was identified as having increased abundance in the iron-treated groups and is known to function as an iron sensor (Table S2) (98, 99).

We observed no change in any of the known elements of CoQ biosynthesis, which is consistent with the Western blot results shown in Fig. 5, *B*–*D*. The analysis also identified 14 proteins with unique abundance changes in the FeSO_4_-only treatment group (Table S3), as well as five proteins that showed altered abundance in both the FeSO_4_-only and PQ-only treatment groups but not in their respective NAC co-treatment groups (Table S4). Although these could be involved in the changes affecting CoQ levels, their identities do not immediately suggest a mechanism. Among these lists, we find three proteins that are particularly interesting to explore in the future. They are Golgi phosphoprotein 3 which plays a crucial role in vesicle trafficking from Golgi to plasma membrane, intermembrane lipid transfer protein VPS13C which is implicated in lipid transfer between membranes as well as in lysosomal homeostasis, and vacuolar protein sorting-associated protein 4B, a component of the endosomal sorting complex required for transport (ESCRT) pathway which is essential for endocytosis and cargo delivery to the lysosome for degradation (100, 101, 102).

## Discussion

We undertook the studies described here to establish whether CoQ trafficking could be targeted to increase CoQ levels. We identified a number of treatments that bring about changes in CoQ levels. Our observations with these treatments suggest that they affect CoQ trafficking and not CoQ synthesis: (1) Iron supplementation in the form of FeSO_4_ leads to a major increase in cellular CoQ, with only a minor effect on CoQ in mitochondria, where CoQ is synthesized, and where therefore an effect on synthesis should be most apparent (Figs. 1 and 2, *A*–*D*). (2) Iron supplementation increases the accumulation of exogenous CoQ (Fig. 3). Obviously, this effect cannot be attributed to endogenous synthesis. (3) PQ treatment leads to increased overall CoQ levels with no observable increase in mitochondrial levels (Fig. 4, *C* and *D*). It is difficult to envision, in any simple way, a mechanism that acts on the rate of synthesis to increase overall CoQ in a major way without affecting CoQ in the compartment in which it is synthesized. (4) Inhibition of aspects of lysosomal lipolysis with Lalistat 1 or inhibition of lysosome acidification with Baf-A1 led to increased cellular CoQ.

In addition, we observed no change in the abundance of several proteins essential for CoQ synthesis (Figs. 5*B* and S6). In fact, the level of COQ7 was decreased with increased iron supplementation. Iron did alter something in the expression, degradation, or post-translational processing of COQ4 (Fig. 5*C*). However, PQ treatment did not, although PQ treatment also resulted in a major increase in cellular CoQ (Fig. 5*D*). Whether the COQ4 protein band change detected after iron exposure corresponded to a functionally relevant change affecting CoQ synthesis is unknown at this time.

How CoQ is distributed to other membranes following synthesis in the mitochondria remains largely unexplored. Studies in yeast suggest that the bridge-like structure that forms at the membrane contact site between the endoplasmic reticulum (ER) and the outer mitochondrial membrane is necessary for CoQ export from the mitochondria (103). An *in vitro* study that followed the appearance of newly synthesized CoQ_10_ in mammalian cells also suggested that the endomembrane system might be involved in CoQ distribution (104). More recently, STARD7, a lipid-binding protein that is known to be required for phosphatidylcholine delivery to the mitochondria, was shown to play a role in CoQ transport to the plasma membrane (105). Autophagic degradation of mitochondria (known as mitophagy) could release CoQ into lysosomes, which may ultimately lead to its elimination. Moreover, lysosomes interact with the Golgi apparatus and the endosome through vesicle trafficking (106). Since these membranes are likely to contain CoQ, these could also be the pathways that are taken to bring CoQ to the lysosome (107). We found that both Lalistat 1 and Baf-A1, which act on lysosome function, increased cellular CoQ levels (Figs. 6, *A* and *B* and S7*A*). Baf-1A, by inhibiting V-ATPase-dependent lysosomal acidification, inhibits both lysosomal function and the fusion between autophagosomes and lysosomes. Its effect on the level of CoQ could be secondary to defective autophagic flux and impaired lysosomal degradation. Lalistat 1 is a competitive inhibitor of lysosomal acid lipase (LAL), the only intracellular lipase known to be active at acidic pH. They are known to hydrolyze cholesteryl esters and triglycerides that are delivered through endocytosis or from the membranes of the organelles (*e.g.*, lipid droplet) *via* autophagy, releasing fatty acids and free cholesterol (108, 109). Pharmacological LAL inhibition can lead to lipid buildup in lysosomes, causing lysosome swelling and impairing their normal function, which can also reduce autophagy flux (88, 110, 111). Thus, the observed effect of Lalistat 1 on CoQ is likely due to the disruption of lysosome-mediated degradation pathways. Future studies will be needed to establish whether the observed effects of Baf-A1 and LAL inhibition on CoQ indeed result from disrupting the lysosome-dependent nonselective bulk degradation of intracellular membranes or if a more specific mechanism directly regulating CoQ turnover exists. In other words, lysosomes might influence CoQ metabolism through general catabolic processes, or they might be involved in CoQ turnover in a more specific way.

It is of interest to mention that altered CoQ levels have been noted in association with lysosomal disorders. Reduced plasma CoQ_10_ levels have been reported in patients with mucopolysaccharidoses (MPS), a group of lysosomal storage disorders caused by deficiencies in enzymes needed for the degradation of glycosaminoglycans (112). Gaucher fibroblast cell lines harbouring the L444P/L444P mutation in the GBA1 gene, which encodes lysosomal acid β-glucocerebrosidase, were shown to have a 24% reduction in total CoQ content (113).

Detection of CoQ breakdown products from the urine and feces of animals after administration of exogenous CoQ found that the main products have an intact quinone ring (114, 115). Outside mitochondria, the lysosome is one of the organelles that contains relatively high amounts of CoQ (8). The lysosome contains more than 60 hydrolase enzymes (*e.g.*, lipases, cathepsins, nucleases, glycosidases) that target different groups of biomolecules, typically peptides, glycosidic bonds, natural lipids, and nucleic acids, thereby responsible for the organelle’s degradative activity (106, 116). Most likely, however, they do not directly hydrolyze CoQ or its isoprenoid side chain, which could be delivered to the lysosomal lumen *via* endocytosis, autophagy, or phagocytic pathways. In fact, so far, no dedicated CoQ/isoprenoid degradation enzymes have been reported in eukaryotes. However, cells lose CoQ rather quickly after biosynthesis is inhibited (61, 62, 63, 64). Thus, if indeed there is no enzyme capable of degrading CoQ or isoprenoids, one has to speculate that CoQ must be lost from the cells by secretion and ultimate elimination through some exocrine secretion, such as bile, in which CoQ has indeed been detected (115).

As mentioned above, the effect of excess iron and PQ on CoQ levels is likely to involve a redox-related mechanism. Lysosomes are considered to be vulnerable to the deleterious effects of ROS because they lack antioxidant enzymes and are particularly vulnerable to membrane permeabilization since their functions depend on an acidic intraluminal pH (113, 117, 118). Furthermore, because much of iron recycling takes place in the lysosome through the continuous breakdown of iron-containing organelles (such as mitochondria) and metalloproteins (like transferrin), the lysosome accommodates a relatively large pool of redox-active iron (*i.e.*, Fe^2+^) (119, 120). However, the small but significant additive effect of Baf-1A and iron on CoQ levels, along with their differential effects on the biosynthetic intermediate DMQ and the lack of effect of iron on the autophagosome-associated form of LC3 (LC3-II), suggests that iron supplementation affects cellular CoQ levels mostly through a lysosome-independent mechanism (Figs. 6, *B* and *C* and S7*B*).

It is interesting to consider the possibility that a proportion of CoQ molecules that reach the lysosome by autophagy or other mechanisms can be removed from the lysosomal lumen and deposited into the lysosomal limiting membrane, from which they are recycled to other organelles rather than being eliminated - the fate observed for a number of lipids (116, 121). An increase in recycling without a decrease in synthesis would lead to a higher cellular concentration. We speculate that certain artificial stimuli, such as iron supplementation or PQ, might lead to increased CoQ recycling with little negative feedback on the regulation of synthesis. That this might be the case is reinforced by the observation that loading of exogenous CoQ_10_ did not lead to a significant decrease in the levels of endogenous CoQ (Fig. 3) (51, 65, 122, 123).

## Conclusions

Taken together, our study presents evidence that cellular CoQ levels can be elevated by mechanisms that don’t involve increased synthesis. The mechanisms appear to involve a redox process, as the elevation of CoQ levels produced by iron and PQ is suppressed by NAC. Yet, these mechanisms do not require an overall increase in oxidative stress. We also found evidence suggesting a key role of lysosomes in regulating CoQ levels. The detailed molecular mechanisms and functional consequences of the elevation of CoQ level by interfering with the fate of CoQ following its mitochondrial synthesis remain to be characterized.

## Data availability

All data are available upon request to Siegfried Hekimi at siegfried.hekimi@mcgill.ca.

## Supporting information

This article contains supporting information.

## Conflict of interest

The authors declare that they do not have any conflicts of interest with the content of this article.
