# Supplementary material for: Elevated cellular accumulation of endogenous and exogenous CoQ by altered intracellular trafficking
Source: J Biol Chem. 2025 Nov 4;301(12):110878. doi: 10.1016/j.jbc.2025.110878 (PMC12702331; doi:10.1016/j.jbc.2025.110878)
Supplement: Supporting Information [file mmc1.pdf]

Supplementary Materials and Methods

Seahorse Extracellular Flux Analysis

Cellular oxygen consumption rates (OCR) were measured using the Seahorse Bioscience XFe96 Extracellular Flux Analyzer (Agilent Technology) as previously described [6]. Briefly, RAW264.7 cells were seeded into XFe96 Pro cell culture microplates and treated with $FeSO_4$ for 2 days before the measurement. OCR were recorded under the baseline condition, followed by the injections of oligomycin (1 µg/µg), Carbonyl cyanide p-trifluoromethoxyphenylhydrazone (FCCP) (2.5 µM) and antimycin A (5 µM) plus rotenone (0.5 µM). Final OCR values were normalized to total protein content per sample as determined by the BCA assay (Thermo Scientific).

Cytotoxicity LDH Assay

The LDH assay was carried out using CytoTox 96 Non-Radioactive Cytotoxicity Assay Kit (Promega) following the manufacturer's instructions. Total release of intracellular LDH after cell lysis with Triton X-100 was set as the maximal LDH release. Results are expressed as a percentage of this maximal LDH release.

Crystal violent staining

Quantification of cell viability using Crystal violet staining was performed as previously described [1]. Briefly, after staining of live cells in the wells, the bound dye was solubilized with 10% acetic acid, and absorbance was measured at 590nm using a plate reader (TECAN Infinite M1000).

Supplementary Tables

Table S1. Measured CoQ concentrations, expressed as ng/mg protein, in the control/no-treatment groups in the figures presenting CoQ levels as relative percentages normalized to the no-treatment controls. These values were set as 100% in those figures for normalization across the figures. Data are expressed as mean ± SEM. NR: not reported. Mito.: mitochondria.

Figure	Cell	CoQ concentration (ng/mg protein)	
		CoQ_9	CoQ_{10}
Figure 1A	RAW264.7	193.9 ± 13.0	NR
Figure 1B	MEFs	57.5 ± 1.7	NR
Figure 1C	HeLa	NR	24.8 ± 0.6
Figure 2A	RAW264.7	168.9 ± 13.0	9.2 ± 0.7
Figure 2B	RAW264.7	189.0 ± 7.9	NR
Figure 2C	RAW264.7	1231.8 ± 4.1 (Mito.)<br>41.8 ± 2.2 (whole cell)	NR
Figure 2D	RAW264.7	181.3 ± 4.3	NR
Figure 4A	RAW264.7	145.2 ± 4.4	NR
Figure 4C	RAW264.7	149.4 ± 2.2	NR
Figure 4D	RAW264.7	1008.3 ± 41.3 (Mito.)<br>39.2± 1.5 (whole cell)	NR
Figure 6A	RAW264.7	157.1 ± 5.3	NR
	Hela	NR	33.2 ± 1.6
Figure 6B	RAW264.7	163.7 ± 8.7	NR
	Hela	NR	31.2 ± 4.1

Table S2. Differentially expressed proteins after $FeSO_4$ treatment identified by proteomics.

UniProtKB ID	Protein Name	Gene Name	Expression
P29391	Ferritin light chain 1	*Ftl1*	Overexpressed in both $FeSO_4$ and $FeSO_4$+NAC group
P09528	Ferritin heavy chain	*Fth1*	
P57716	Nicastrin	*Ncstn*	
A1L314	Macrophage-expressed gene 1 protein	*Mpeg1*	
P10649	Glutathione S-transferase Mu 1	*Gstm1*	
Q8BX70	Intermembrane lipid transfer protein VPS13C	*VPS13C*	
Q6P4T2	U5 small nuclear ribonucleoprotein 200 kDa helicase	*Snrnp200*	
P46467	Vacuolar protein sorting-associated protein 4B	*Vps4b*	
Q07417	Short-chain specific acyl-CoA dehydrogenase, mitochondrial	*Acads*	
P28271	Cytoplasmic aconitate hydratase	*Aco1*	
P70398	Ubiquitin carboxyl-terminal hydrolase 9X	*Usp9x*	
Q8BIW1	Exopolyphosphatase PRUNE1	*Prune1*	
P56542	Deoxyribonuclease-2-alpha	*Dnase2*	
Q00519	Xanthine dehydrogenase/oxidase	*Xdh*	
P70188	Kinesin-associated protein 3	*Kifap3*	
Q91VE6	MKI67 FHA domain-interacting nucleolar phosphoprotein	*Nifk*	
Q9D8N0	Elongation factor 1-gamma	*Eef1g*	Underexpressed in both $FeSO_4$ and $FeSO_4$+NAC group
P30416	Peptidyl-prolyl cis-trans isomerase FKBP4	*Fkbp4*	
Q9CZD3	Glycine--tRNA ligase	*Gars1*	
Q80WJ7	Protein LYRIC	*Mtdh*	
Q61033	Lamina-associated polypeptide 2, isoforms alpha/zeta	*Tmpo*	
Q6GQT9	BOS complex subunit NOMO1	*Nomo1*	
Q99KV1	DnaJ homolog subfamily B member 11	*Dnajb11*	
P62267	Small ribosomal subunit protein uS12	*Rps23*	

Table S3. Differentially expressed proteins identified through proteomics that were exclusive to the $FeSO_4$ treatment-only group.

UniProtKB ID	Protein Name	Gene Name	Expression
C0HKG5	Ribonuclease T2-A	*Rnaset2a*	Overexpressed uniquely in the $FeSO_4$-only treatment group
Q9JHU4	Cytoplasmic dynein 1 heavy chain 1	*Dync1h1*	
Q8BG07	5'-3' exonuclease PLD4	*Pld4*	
Q61191	Host cell factor 1	*Hcfc1*	
P28658	Ataxin-10	*Atxn10*	
Q8BFR4	N-acetylglucosamine-6-sulfatase	*Gns*	
Q80UY2	E3 ubiquitin-protein ligase KCMF1	*Kcmf1*	
Q9D1K2	V-type proton ATPase subunit F	*Atp6v1f*	
Q99JT9	Acireductone dioxygenase	*Adi1*	
Q5MJS3	Extracellular serine/threonine protein kinase FAM20C	*Fam20c*	
P62743	AP-2 complex subunit sigma	*Ap2s1*	
Q6PF93	Phosphatidylinositol 3-kinase catalytic subunit type 3	*Pik3c3*	
Q3UFK8	FERM domain-containing protein 8	*Frmd8*	
Q505F5	Leucine-rich repeat-containing protein 47	*Lrrc47*	Underexpressed uniquely in the $FeSO_4$-only treatment group

Table S4. Differentially expressed proteins identified in both $FeSO_4$- and PQ-treated cells without NAC co-treatment.

UniProtKB ID	Protein Name	Gene Name	Expression
Q9CQQ7	ATP synthase F(0) complex subunit B1, mitochondrial	*Atp5pb*	Overexpressed in both the $FeSO_4$-only and PQ-only treatment groups
Q8BIG7	Catechol O-methyltransferase domain-containing protein 1	*Comtd1*	
Q9CRA5	Golgi phosphoprotein 3	*Golph3*	
Q9DBZ5	Eukaryotic translation initiation factor 3 subunit K	*Eif3k*	
Q9D6N5	Dr1-associated corepressor	*Drap1*	

Supplementary Figures

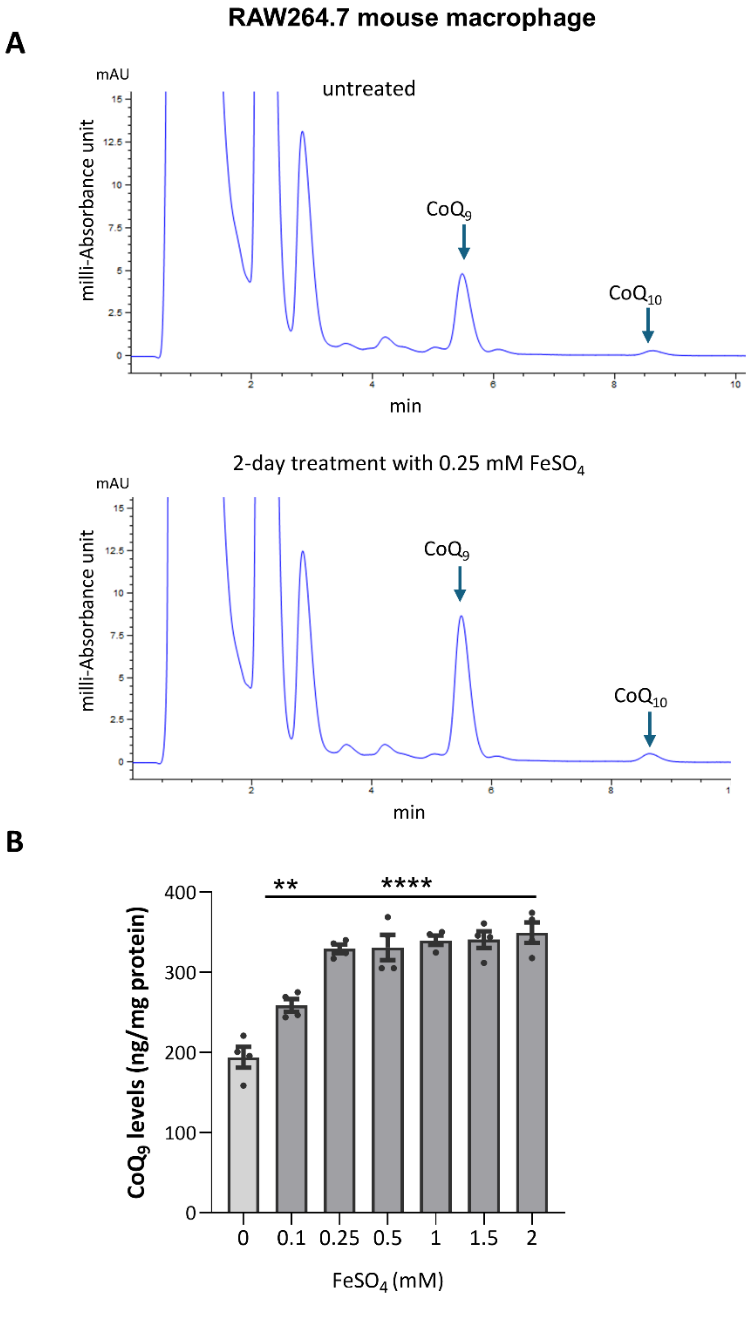

Fig. S1. **Measurement of CoQ levels in RAW264.7 cells following FeSO4 treatment.** (A) HPLC chromatograms of CoQ extracts from RAW264.7 cells treated with or without $FeSO_4$. (B) CoQ_9 levels were normalized to protein amounts. Cell lysates containing equal amounts of protein were used to extract and quantify CoQ after 2-day treatment with or without $FeSO_4$. Both CoQ_9 and CoQ_{10} levels were significantly elevated in the cells exposed to $FeSO_4$. Bars represent mean ± SEM (n=4). $^{**}p<0.01$ and $^{****}p<0.0001$ compared with no treatment control (one-way ANOVA followed by Dunnett's multiple comparison test).

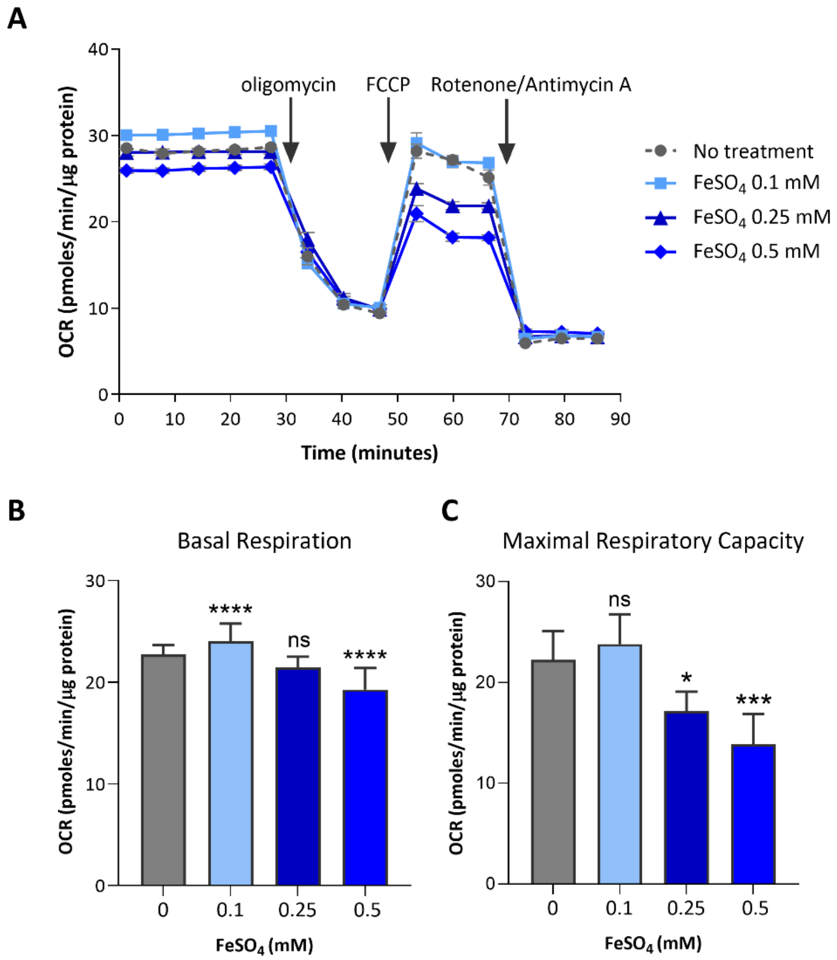

Fig. S2. OCR measurement by Seahorse extracellular flux analysis. RAW264.7 cells were treated with $FeSO_4$ for 2 days before Seahorse mitochondrial stress analysis. (A) Oxygen consumption rate trace was determined using a Seahorse XFe96 Analyzer. The arrows indicate the addition time of oligomycin, FCCP and rotenone/antimycin A. (B) Baseline respiratory rate. (C) Maximum respiratory capacity. Bars represent mean ± SEM. $^{*}p<0.05$, $^{***}p<0.001$, and $^{****}p<0.0001$ compared with no treatment control (one-way ANOVA followed by Dunnett's multiple comparison test).

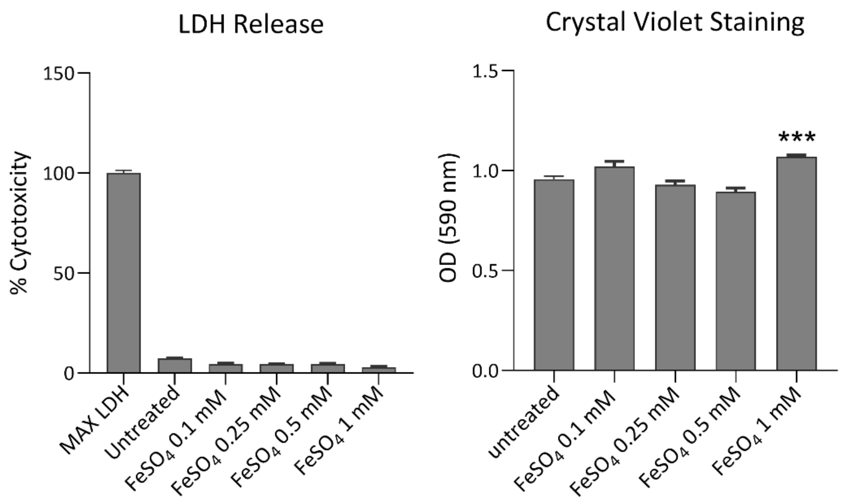

Fig. S3. **Cell viability measurements using the CytoTox 96 Non-radioactive Cytotoxicity Assay kit or crystal violet staining assay in RAW264.7 cells treated with $FeSO_4$ for 2 days.** Data are shown as mean ± SEM (n=6-8). Maximum LDH release control was represented as 100% cytotoxicity, and all samples were normalized to the maximum LDH release control. For the crystal violet staining assay, after staining of the live cells in the wells, the bound dye was solubilized, and the absorbance was read at 590 nm. $^{***}p<0.001$ compared to untreated control (One-way ANOVA followed by Dunnett's post hoc test.

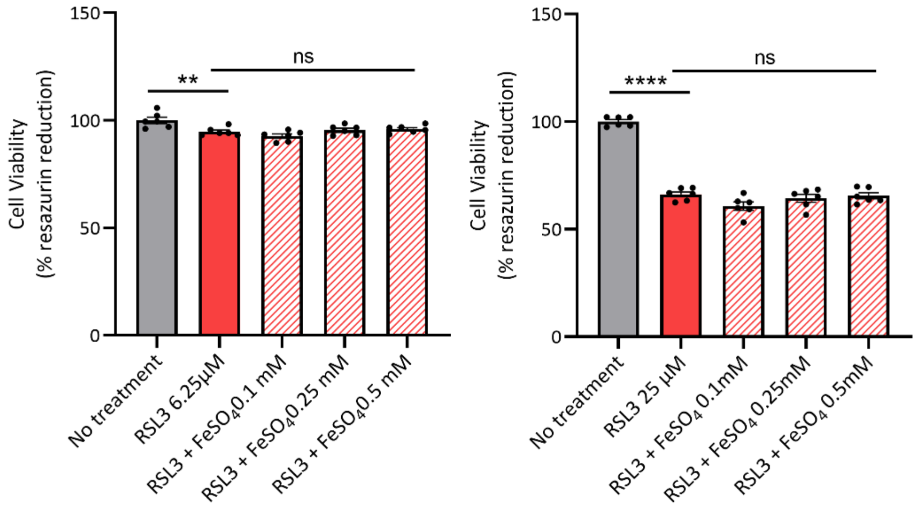

Fig. S4. **Cell viability measument of RAW264.7 cells after treatment with RSL3 alone or in combination with $FeSO_4$.** Viability was measured using resazurin assay after 2 days of the indicated treatments. Data are shown as mean ± SEM (n=6) and expressed as percentages relative to the no-treatment control, which wasset to 100%. ns: no significant, $^{**}p < 0.01$, $^{****}p < 0.0001$ vs. no-treatment control (one-way ANOVA with Sidak's post hoc test or *t*-test).

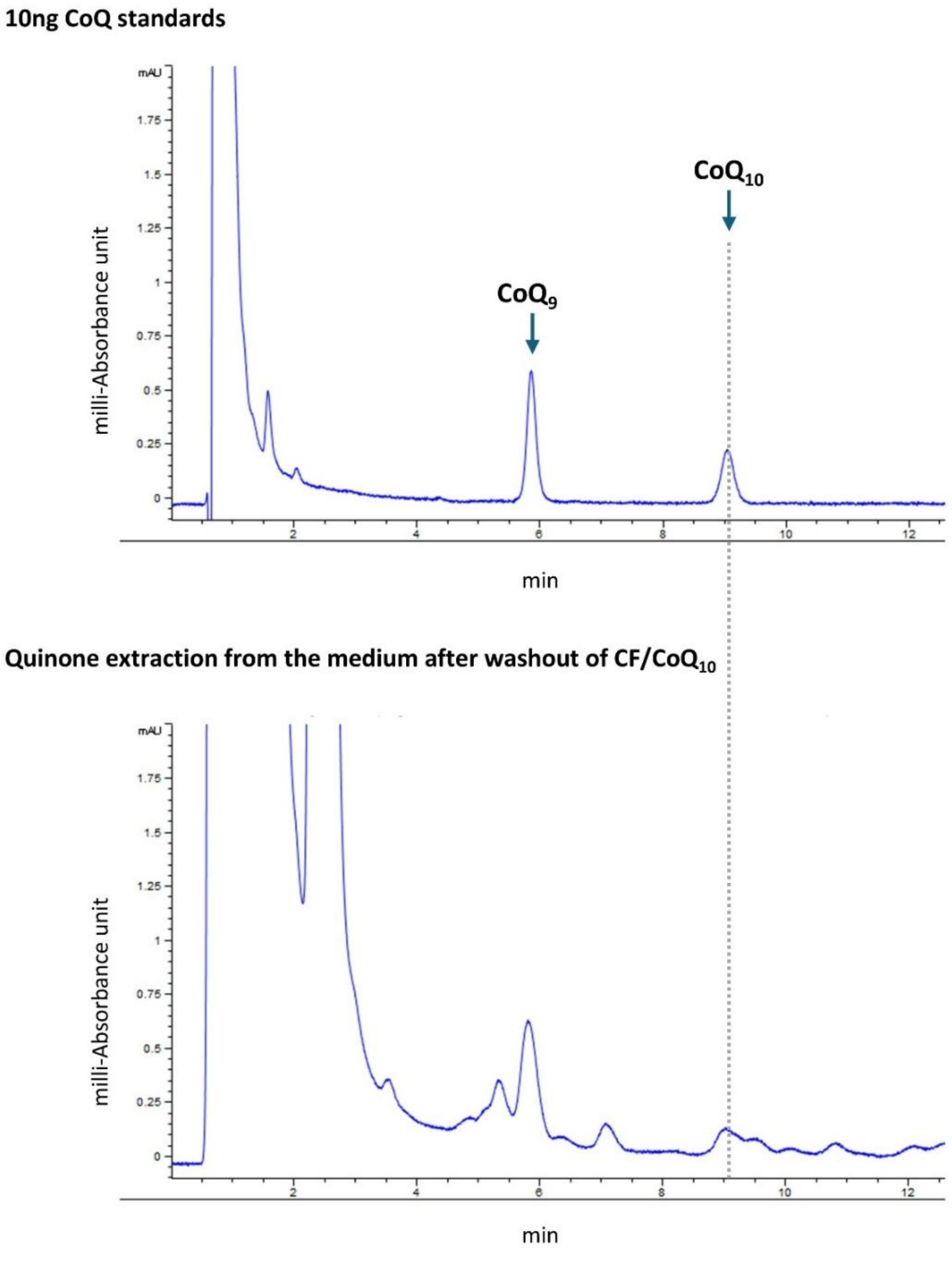

Fig. S5. **CoQ_{10} detection in the culture medium of RAW264.7 cells after washout of CF/CoQ_{10} treatment**. Upper panel shows the HPCL chromatogram of 10 ng of CoQ standards. This corresponds to a CoQ_{10} concentration of 5.8 nM in 2mL of culture medium. The lower panel shows the HPLC chromatogram of quinone extracted from 2ml of culture medium after washing out CF/CoQ_{10} from the medium of RAW264.7 following 1 day of treatment at 2.5 μM.

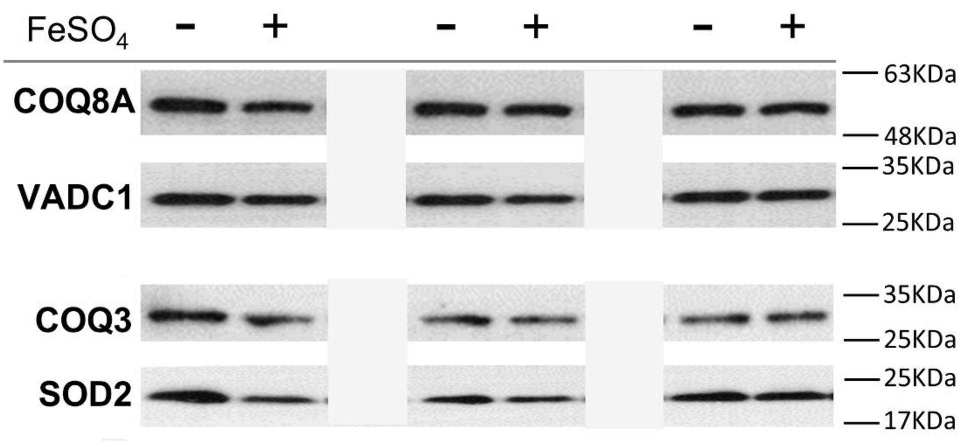

Fig. S6. **Image of Western Blot for COQ8A and COQ3 protein levels in RAW264.7 cells treated with or without $FeSO_4$.** Cells were harvested for Western Blot after 2 days of treatment with or without $FeSO_4$. Loading controls used are VDAC1/Porin and SOD2. Blots obtained from three independent experiments are shown.

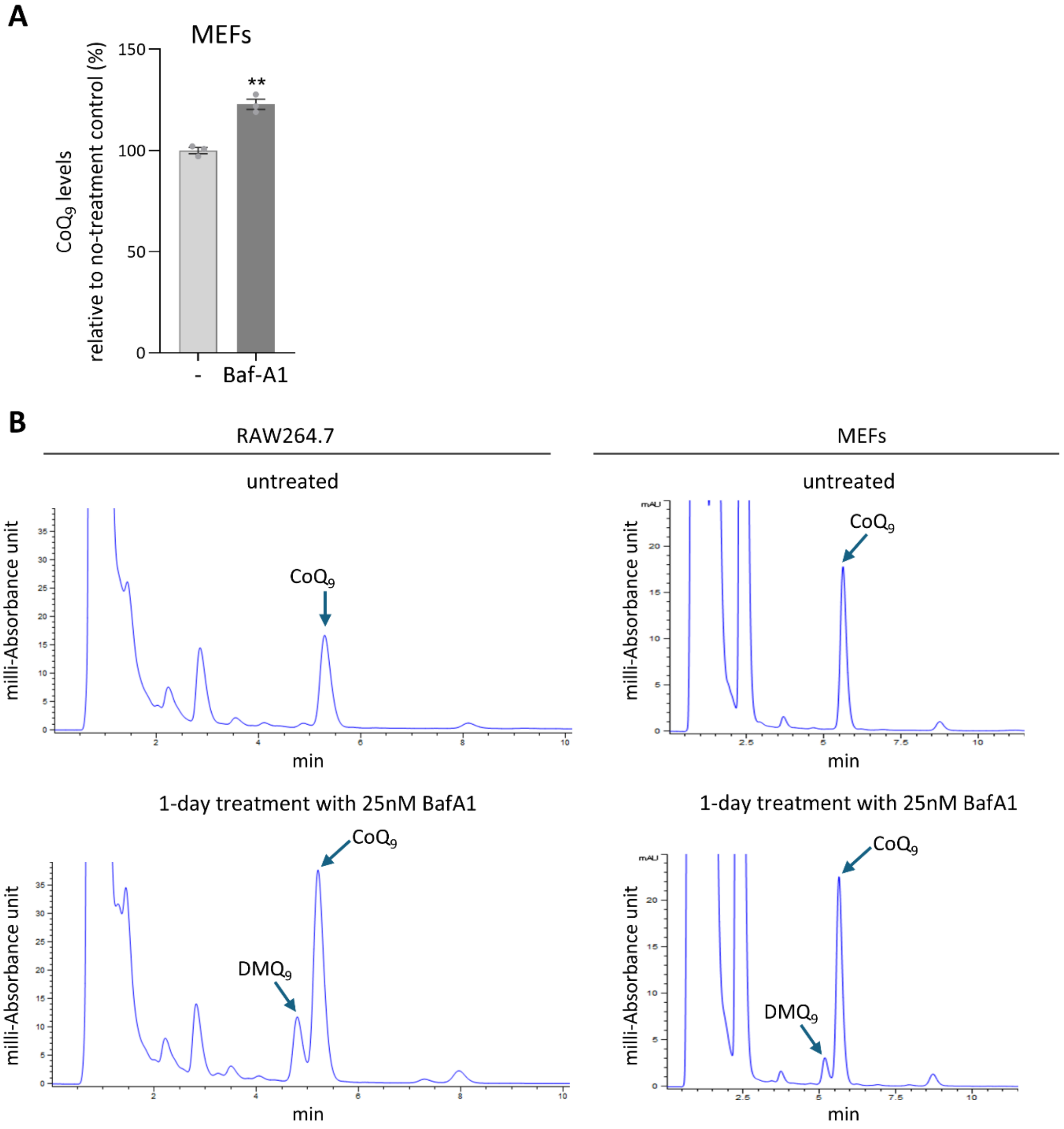

Fig. S7. **Effects of Bafilomycin A1 (Baf-A1) on cellular CoQ.** (A) CoQ quantification in mouse embryonic fibroblasts (MEFs) treated with or without Baf-A1 for 1 day. Data are shown as mean ± SD (n=3). CoQ levels were normalized to protein content and expressed as a percentage relative to the DMSO-treated control (0.1% v/v), which was assigned a value of 100%. $^{**}p < 0.01$, unpaired Student *t*-test. (B) HPLC chromatograms of quinone extracts from RAW264.7 macrophages or MEFs treated with or without Baf-A1. Cell lysates containing equal amounts of protein were used to extract and quantify CoQ. In addition to the elevation of CoQ levels, accumulation of the biosynthetic intermediate demethoxyquinone (DMQ) was observed in Baf-A1-treated cells.

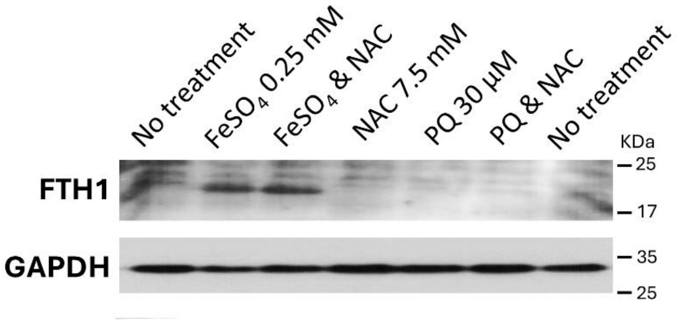

Fig. S8. **Western Blot analysis of Ferritin Heavy Chain 1 (FTH1)**. RAW264.7 cells were collected for analysis 2 days after the indicated treatments. GAPDH was used as a loading control. An elevation of ferritin heavy chain (FTH1) levels was observed in $FeSO_4$ and $FeSO_4$ and N-acetylcysteine (NAC) co-treated cells, but not in paraquat (PQ)-treated cells.
